# The modified weight bias internalization scale: psychometric validation of three versions in a sample of university students

**DOI:** 10.1007/s40519-025-01741-4

**Published:** 2025-03-20

**Authors:** Paul E. Jenkins, Lacin Baysen

**Affiliations:** School of Psychology and Clinical Language Sciences, University of Reading, Reading, RG6 6ES UK

**Keywords:** Internalised weight bias, Confirmatory factor analysis, Measurement, Validation, Students

## Abstract

**Purpose:**

The Modified Weight Bias Internalization Scale (WBIS-M) is perhaps the most frequently used measure of internalised weight bias and has growing support for its psychometric properties. However, there is a lack of clarity regarding how many items are necessary for adequate interpretation of the WBIS-M and limited study of internalised weight bias in young adults. The aims of this study are to evaluate different versions of the WBIS-M, assessing structural and convergent validity.

**Methods:**

The current study recruited 205 university students (aged 18–46, mean body mass index = 22.60 kg/m^2^) in the UK and examined the factor structure, reliability, and convergent validity of the WBIS-M, looking at 11-item, 10-item, and 9-item versions.

**Results:**

Confirmatory factor analysis suggested that a 10-item version of the WBIS-M showed acceptable structural validity and expected correlations with relevant constructs (depression, anxiety, weight status, and eating pathology). Estimates of internal consistency reliability were high for all three versions.

**Conclusion:**

Given potential problems with one item, the 10-item WBIS-M presents a measure of internalised weight bias with sound psychometric properties in young adults.

*Level of evidence*: Level III, well-designed cohort study.

**Supplementary Information:**

The online version contains supplementary material available at 10.1007/s40519-025-01741-4.

## Introduction

Discrimination towards individuals with obesity has long been considered a driver of poorer mental health, impaired quality of life, and social problems in this population [1]. Further, negative attitudes towards weight can impair individuals of different weight statuses, with the potential for stigma to be both received from others and directed towards oneself (e.g., [2]).

Research into internalisation of weight-biased attitudes (weight-bias internalisation, or WBI) has been increasing since the turn of the millennium and suggests that WBI “is consistently associated with negative mental health outcomes such as depression, anxiety, poor self-esteem and body image, disordered eating and impaired mental HRQOL [health-related quality of life]” ([2], p. 1159). Several sociodemographic variables have been linked to higher WBI, and a number of studies have found higher weight bias in younger, compared to older, adults (e.g., [3]), suggesting that this population is at heightened risk for weight-biased attitudes.

Given the influence of WBI on health-related outcomes, various measures have been developed to assess WBI and weight stigma, and a review completed in 2020 [4, 5] found 18 measures intended to assess internalised weight bias, the most commonly used of which was the Weight Bias Internalization Scale (WBIS; [6]). Whilst support for the psychometric properties of the WBIS has generally been found [4], the measure itself is restricted to use with individuals who consider themselves overweight. As a result, Pearl and Puhl [7] modified the wording of the WBIS to cover individuals of all weight statuses and thus assess internalised weight bias regardless of weight classification, a measure known the Modified WBIS, or WBIS-M.

Adding to data on the WBIS, the WBIS-M has been found to demonstrate good psychometric properties (e.g., discriminant validity) and expected correlations with measures of eating pathology, depression, and anxiety (e.g., [7, 8]). In addition to other estimates of validity, the factor structure (i.e., structural validity) of the WBIS-M has been investigated with exploratory (EFA) and confirmatory factor analysis (CFA). In samples of adults, translations of the WBIS-M into Spanish [9], Greek [10], Turkish [11], and Norwegian [12] have offered support for a one-factor, 11-item measure. In a sample of secondary school students in Barcelona, EFA and CFA suggested a one-factor structure of a 10-item version, with one item (Item 1: “Because of my weight, I feel that I am just as competent as anyone”) excluded due to a poor factor loading [13]. A similar study of first-generation Asian immigrants in the United States examined the factor structure of this 10-item version of the WBIS-M, confirming its unidimensionality and also offering “preliminary support for a nine-item version” (also excluding Item 9, “I am OK being the weight that I am,” which is reverse-scored; [14], p. 17). Finally, in a large multinational study [15], the (unidimensional) 11-item WBIS-M was found to evidence poor fit, which was improved by removing Item 1 (as in [13], and [8]) and allowing some residual (error) variances to correlate (see also [12]).

Whilst results from these studies offer support for the unidimensionality of the WBIS-M, findings are inconsistent (see also [2]) and there are limitations to both the samples and methods used. There have been limited studies using English language versions, and few with young adult and university samples, who may present with stronger internalised weight bias. One (unpublished) work has reported CFA of the 11-item WBIS-M with a UK sample [16], which comprised young adults (mean age = 26.0 years), around 75% of whom were university students. The fit indices (Table C1 in [16]) suggest near-adequate fit on some measures, but poor fit on another, leading to uncertain conclusions regarding structural validity in such samples.

Given inconsistent findings regarding the structural validity of the 11-item version [9–16], few studies in English-speaking countries and the UK in particular, and limited support for reduced-item measures, the current study aims to compare three hypothesised factor structures of the WBIS-M—specifically, 11-item, 10-item, and 9-item [14] versions. Further, the study will look at associations with variables known to be correlated with WBI (i.e., depression, anxiety, and eating pathology) across all models.

## Methods

### Participants

Two-hundred and five undergraduate and postgraduate students were recruited from a moderately large UK university through local advertising (posters on campus) and the Psychology Research Participation Scheme. Data collection took place online, including providing informed consent, and the study was approved by the University’s Ethics Committee.

### Measures

Participants were asked to provide demographic information (age, gender, weight, height, ethnicity; see Table 1), in addition to responses to several questionnaires. The *WBIS-M* [7] consists of 11 items rated on a 7-point Likert scale (from ‘Strongly Disagree’ to ‘Strongly Agree’). Items are averaged to produce a total score, with higher scores indicative of greater internalised weight bias. The 8-item *PHQ-8* assesses symptoms of depression using a 4-point Likert scale (from ‘Not at all’ to ‘Nearly every day’ over the past 2 weeks), and a total score indicates more severe symptoms [17]. The 7-item *GAD-7* assesses symptoms of anxiety, also using a 4-point Likert scale (from ‘Not at all’ to ‘Nearly every day’ of the past 2 weeks). Akin to the PHQ-8, a Total score indicates more severe symptoms [18]. The 26 items of the *Eating Attitudes Test* (EAT) can be used as a measure of eating disorder symptoms, using a 6-point Likert scale (“Always” to “Never”), whereby higher scores indicate more frequent disordered eating attitudes [19].

**Table 1:** Demographics of the sample

Characteristic	Descriptive data
Gender, n (%)	
Female	172 (83.9)
Male	25 (12.2)
Non-binary	3 (1.5)
Opted not to say	5 (2.4)
Age, M (SD), range	20.48 (3.25), range = 18–46
BMI, M (SD), range	22.60 kg/m^2^ (4.84), range = 13.7–45.8
Weight status, n (%)	
Underweight (BMI < 18.5 kg/m^2^)	29 (14.2)
Normal weight (BMI 18.5–24.9 kg/m^2^)	133 (64.9)
Overweight (25.0–29.9 kg/m^2^)	21 (10.2)
Obese (BMI > 30.0 kg/m^2^)	19 (9.3)
Missing	3 (1.5)
Ethnicity	
Asian or Asian British	44 (21.5)
Mixed or Multiple ethnic groups	15 (7.3)
White	125 (61.0)
Black British, Caribbean or African	7 (3.4)
Other	3 (1.5)
Missing	11 (5.4)

### Statistical analysis

Analyses were conducted using R (v. 4.3.0; [20]). For CFA, the *lavaan* package (0.6–17, [21]) was used, and the *psych* package [22] for estimates of skewness, kurtosis, sampling adequacy (Kaiser–Meyer–Olkin measure; KMO), and factor analysis reliability (using ρ_FA_^1^; [23, 24]). The *correlation* package [25] was used for correlations.

To identify models within CFA, factor variances were fixed to 1 and items were treated as categorical (see [21]). Mardia’s test suggested the presence of non-normality, so robust estimation (Weighted Least Squares Means- and Variance-adjusted; WLSMV) was used in CFA. Skewness estimates for WBIS-M items ranged from −0.30 (Item 3) to 1.28 (Item 8). To assess model specification, common fit indices (comparative fit index [CFI], Tucker–Lewis index [TLI], root mean square error of approximation [RMSEA, including 90% CIs], standardized root mean squared residual [SRMR]) were used (see [26]). Recent work has suggested that the RMSEA is sensitive (and more likely to suggest rejection of models) in cases of strong factor loadings (i.e., λs = 0.70–0.90), although this phenomenon will also affect CFI, and SRMR, albeit to a lesser extent (e.g., [27, 28]). As such, whilst the RMSEA is reported, we will interpret the findings with this possibility in mind, given reasonably strong factors loadings (typically λs > 0.65) predicted for the WBIS-M (e.g., [9, 10, 13]).

To assess the relationship between WBI and other constructs of interest, non-parametric (Spearman’s *r*) correlations were conducted among the sum scores of the WBIS-M (Total scores based on each model’s items) and PHQ-8, GAD-7, and EAT scores. Participants’ body mass index (BMI; kg/m^2^) was calculated from self-reported weight and height, and used as a further criterion variable.

A sample size of at least 200 was planned, given guidance on conducting CFA and the magnitude of expected item loadings [29, 30]. This figure is also sufficient for correlation analyses (given previous estimates of *r* ≈ 0.45; [7]).

## Results

The overall KMO statistic for WBIS-M items was 0.93 (range = 0.78–0.97), suggesting that data were appropriate for factor analysis. Of note, Item 1 represented the lowest value, with the next lowest being 0.91; this reflects the pattern of (standardised) factor loadings (λs), which were strong (> 0.75) except for Item 1 (see Table 2). Inter-item correlations for the WBIS-M are provided in Online Resource 1. No item on the WBIS-M had more than one missing data point (overall mean = 0.003%), so data were deleted listwise where necessary.

**Table 2:** Fit indices, factor loadings, internal reliability estimates, means, standard deviations, and correlations for the three models of the WBIS-M tested

	11-item WBIS-M	10-item WBIS-M	9-item WBIS-M
Fit indices			
χ^2^	184.40	186.76	128.33
df	44	36	27
CFI	0.91	0.92	0.93
TLI	0.89	0.90	0.91
RMSEA (90% CIs)	0.144 (0.125–0.164)	0.153 (0.132–0.175)	0.156 (0.132–0.182)
SRMR	0.043	0.037	0.038
Factor loadings (λ)			
Item 1	0.29	–	–
Item 2	0.86	0.87	0.87
Item 3	0.86	0.87	0.87
Item 4	0.87	0.88	0.86
Item 5	0.87	0.88	0.88
Item 6	0.95	0.95	0.95
Item 7	0.75	0.75	0.75
Item 8	0.77	0.77	0.77
Item 9	0.80	0.80	–
Item 10	0.81	0.81	0.81
Item 11	0.88	0.88	0.88
ρ_FA_	0.95	0.96	0.96
Total score, mean (SD)	3.71 (1.15)	3.64 (1.29)	3.58 (1.58)
Correlations*			
PHQ-8	0.45	0.46	0.45
GAD-7	0.39	0.40	0.40
EAT	0.58	0.59	0.59
BMI	0.37	0.36	0.37

Item numbers refer to those as first presented for the WBIS-M [7]. *ρ*_*FA*_ factor analysis reliability, *BMI* body mass index, *CFI* comparative fit index, *RMSEA* root mean square error of approximation, *SRMR* standardised root mean square residual, *TLI* Tucker-Lewis index. *All correlations significant at p < 0.001

### Model fit

Robust fit indices for the 10-item and 9-item models were improved over the 11-item model (Table 2). Values fell within the ‘acceptable’ range for the CFI and TLI (i.e., between 0.90 and 0.95), and were ‘good’ for the SRMR (< 0.08; [26]). Regarding the RMSEA, values indicated poor fit, and strong factor loadings of the WBIS-M were seen; specifically, the highest factor loading of the 10-item WBIS-M in this sample was 0.93, with an average of 0.84.^2^ Factor analysis reliability estimates were ≥ 0.95 for the three versions of the WBIS-M (see Table 2).

### Correlations

Correlations with relevant criterion variables (i.e., PHQ-8, GAD-7, EAT, BMI) were all significant (*p*s < 0.001, two-tailed) and of a similar magnitude regardless of which version of the WBIS-M was used (see Table 2).

## Discussion

This study examined the factor structure and psychometric properties of the WBIS-M in a sample of university students in the UK, supporting previous work in other samples demonstrating acceptable fit of a one-factor model. However, whilst fit for the 11-item model was marginal, poor factor loadings for Item 1 suggest that use of the 10-item version of the WBIS-M is more defensible. Given existing work in samples from different backgrounds (e.g., [12–15]) and few notable drawbacks, use of the 10-item WBIS-M to assess WBI should be encouraged.

In addition to structural validity, the 10-item WBIS-M demonstrated expected correlations with depression, anxiety, and eating pathology. The WBIS-M also showed moderate correlations with BMI in this student sample, in contrast to use of the ‘original’ WBIS [6, 7]. Taken together, a number of findings support the convergent validity of the WBIS-M (e.g., [10, 13]), with the current study suggesting that this is the case regardless of which version is used. Removal of Item 9 [14], however, does not seem to confer particular advantages in terms of model fit, at least in this UK student sample. The proportion of missing data for the WBIS-M was very low, in line with previous work [14, 31].

Examining findings regarding structural validity, RMSEA values were above recommended cutoffs for all versions of the WBIS-M and might appear to indicate poor fit, particularly for the reduced-item models. However, recent empirical work has suggested that the RMSEA is very likely to indicate poor fit when factor loadings are high (see [27, 28]), a difficulty exacerbated as removal of one item is based largely on its weak factor loading (in the current study, λ_Item 1_ = 0.29), as well as small correlations with other items. Similarly, some fit indices may be more likely to indicate poor model fit when the proportion of missing data is low [32]. Thus, as often recommended (e.g., [28, 29]), interpretation of fit indices should consider the context and complexity of the models under study rather than adhering strictly to a given ‘cutoff’. In the current study, therefore, the fit indices reported can be taken to indicate good fit of all models given the strong factor loadings, and good performance on the SRMR in particular. However, as Item 1 represents a clear exception, it seems both logical and empirically supported (e.g., [13, 15]) to omit this question from the WBIS-M in future work. Future psychometric studies of the WBIS-M should therefore consider the strong factor loadings typically seen, the small degrees of freedom, and the often-observed data completeness.

A recent study [33] proposed a short form of the WBIS-M, comprising three items (“I feel anxious about my weight because of what people might think of me”, “Whenever I think a lot about my weight, I feel depressed”, “I hate myself for my weight”). Results suggested that this measure can be interpreted similarly regardless of gender, age, and weight status [33], and the suggested factor structure has also been supported in a sample of Lebanese adults – using an Arabic version of the WBIS-3 [34]. However, as a structural equation model with only three indicators and one latent variable, it has zero degrees of freedom and is thus ‘just-identified’; model fit cannot be tested with standard CFA approaches and necessitates different methods (e.g., see [35]) and was therefore not evaluated in the current study.

## Strengths and limits

This is one of only a few studies assessing the structural validity and psychometric properties of the English-language WBIS-M and, additionally, directly compares the performance of three different versions. Commonly used and well-validated measures were used to assess construct validity and findings suggest that one item of the WBIS-M may be (statistically) redundant and removed without compromising key advantages of the questionnaire.

Whilst there is good reason to support the interpretation of the findings based on fit indices (e.g., [26–29, 32]), there are some inconsistencies, particularly when compared to relatively close-fitting models reported in the literature (e.g., CFI of 0.977 [14] and Goodness-of-Fit Index of 0.989 [9]; cf. [12]). Further study, ideally with larger samples, modern methods, and diverse groups might help clarify this. Similarly, whilst the sample size of the current study was adequate for CFA, this might have affected interpretation of some fit indices [30], and small subgroups (e.g., men) did not afford testing of measurement invariance of the WBIS-M. Finally, convergent validity could have been further assessed through inclusion of measures assessing weight stigma or WBI.

## What is already known on this subject?

The WBIS-M has shown acceptable psychometric properties across several international samples. However, there has been limited assessment of a 10-item version of this measure, few empirical comparisons of different versions, and a dearth of work with university students, who are at elevated risk for internalised weight bias.

## What this study adds

The current study offers further support for the unidimensionality of the WBIS-M in a UK student sample, suggesting that a 10-item version shows sound psychometric properties and excludes one item which evidences poor relationships to the overall score. The findings show that internalised weight bias is strongly related to depression, anxiety, eating pathology, and BMI.

## Supplementary Information

Below is the link to the electronic supplementary material.

Supplementary material 1.

## Data Availability

Data supporting this study are available from the University of Reading Research Data Archive at 10.17864/1947.001395.

## Footnotes

1 This statistic is also referred to as *McDonald’s omega* [ω] and used as an indicator of internal consistency reliability; see [23] for a discussion.

2 To illustrate this apparent paradox, Saris et al. [27] found that manipulating factor loadings from 0.70 to 0.85 across a consistent model resulted in a change in RMSEA from 0.00 (indicating near-perfect fit) to 0.092 (representing poor fit, by the Hu & Bentler [26] criteria); Saris et al. conclude that this is “very inconvenient… because the better the measurement model – high loadings – the higher probability of [the model] getting rejected” (p. 569).
