# Supplementary material for: The modified weight bias internalization scale: psychometric validation of three versions in a sample of university students
Source: Eat Weight Disord. 2025 Mar 20;30(1):28. doi: 10.1007/s40519-025-01741-4 (PMC11926019; doi:10.1007/s40519-025-01741-4)

**The Modified Weight Bias Internalization Scale: Psychometric validation of three versions in a sample of university students**

Paul E. Jenkins* & Lacin Baysen

**Affiliations**

School of Psychology and Clinical Language Sciences, University of Reading, Reading. RG6 6ES. United Kingdom

*Corresponding author: School of Psychology and Clinical Language Sciences, University of Reading, Reading. RG6 6ES. E-mail: [pej106@gmail.com](mailto:pej106@gmail.com)

Supplementary Figure 1. Inter-item correlations for the WBIS-M


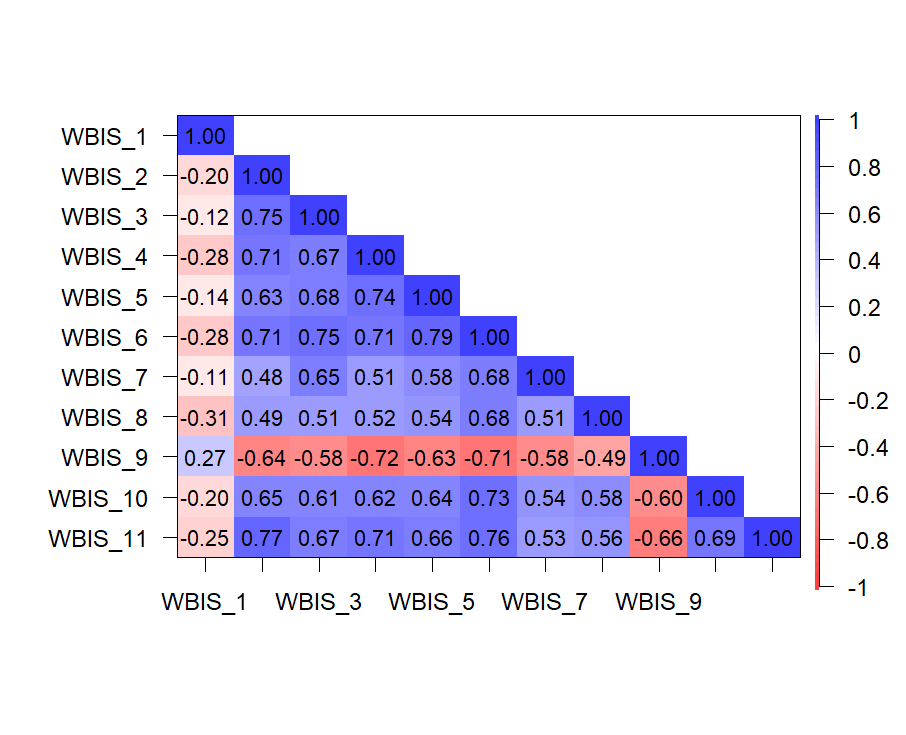

Supplement: Supplementary file 1 — Supplementary material 1. [file 40519_2025_1741_MOESM1_ESM.docx]
